# The impact of hospital revenue on the increase in Caesarean sections in Norway. A panel data analysis of hospitals 1976-2005

**DOI:** 10.1186/1472-6963-11-267

**Published:** 2011-10-12

**Authors:** Jostein Grytten, Lars Monkerud, Terje P Hagen, Rune Sørensen, Anne Eskild, Irene Skau

**Affiliations:** 1 Department of Community Dentistry, University of Oslo, Oslo, Norway; 2 Department of Economics, BI Norwegian Business School, Oslo, Norway; 3 Department of Gynecology and Obstetrics, Akershus University Hospital, Lørenskog, Norway; 4 Department of Health Management and Health Economics, University of Oslo, Oslo, Norway; 5 Institute of Clinical Medicine, University of Oslo, Oslo, Norway

## Abstract

**Background:**

There has been a marked increase in the number of Caesarean sections in many countries during the last decades. In several countries, Caesarean sections are carried out in more than 20 per cent of births. These high Caesarean section rates give cause for concern, both from an economic and a medical perspective. A general opinion among epidemiologists is that the increase in the number of Caesarean sections during the last decade has been greater than could be expected in relation to medical risk factors. Therefore, other explanations must be sought. We studied one potential explanation; the effect that the increase in hospital revenue per bed during the period 1976-2005 has had on the Caesarean section rate in Norway. During this period, hospital revenue increased by about 260% (adjusted for inflation).

**Methods:**

The analyses were carried out using data from the Medical Birth Registry 1976-2005 from Norway. The data were merged with data about hospital revenue, which were obtained from Statistics Norway. The analyses were carried out using annual data from 46 hospitals. A fixed effect regression model was estimated. Relevant medical control variables were included.

**Results:**

The elasticity of the Caesarean section rate with respect to hospital revenue per bed was 0.13 (p < 0.05). This represents an increase in the Caesarean section rate from the basis year 1976 to the final year 2005 of about 35 per cent. Most of the variables measuring characteristics of the health status of the mother and child had the expected effects.

**Conclusion:**

The increase in hospital revenue explains only a small part of the increase in the Caesarean section rate in Norway during the last three decades. The increase in the Caesarean section rate is considerably greater than could be expected, based on the increase in hospital revenue alone. The strength of our study is that we have estimated a cause and effect relationship. This was done by using fixed effects for hospitals, a lagged revenue variable and by including an extensive set of control variables for the risk factors of the mother and the baby.

## Background

There has been a marked increase in the number of Caesarean sections in many countries during the last few decades [1-5]. For example, Caesarean sections are now performed in over 22 per cent of all births in Great Britain [2] and in 30 per cent of all births in the USA [3,6]. The proportion in the Nordic countries is slightly lower - just under 20 per cent [7]. In all the Nordic countries, the Caesarean section rate was about 5 per cent or lower at the beginning of the 1970s. Caesarean section is now the most common major surgical procedure for women in the USA [3,6]. With this great increase in the number of Caesarean sections, the cost of maternity care has also markedly increased. Henderson et al. have done a systematic review of all published studies on the costs of alternative modes of delivery up to 1999 [8]. Altogether they identified 975 studies. Only 49 of these studies fulfilled the inclusion criteria for their review. For these studies the cost of a Caesarean section was in the range £1238 - £8273. The cost for a normal delivery was £629 - £5012. These findings imply that the cost of a Caesarean delivery is about twice the cost of a normal delivery.

It has been claimed that the Caesarean section rate is too high, and that future increase in the rate should be limited, for both economic and medical reasons. For example, in the WHO guidelines, it is recommended that the Caesarean section rate should not be more than 10-15 per cent in any country [9,10]. This is supported by studies that show an increased risk of adverse side effects with Caesarean section, particularly if the medical indications are weak [11-13]. For example, there is an increased risk of stillbirth in the next pregnancy, reduced fertility, and allergy or asthma in the child [12,14-18].

At present, there are no adequate explanations for why the Caesarean section rate has increased so markedly. Epidemiologists have studied whether the increase can be explained in relation to risk factors, but have found little support for this theory [19-22]. The prevalence of several of the medical conditions that are indications for Caesarean section (for example failure of development of the foetus, abnormal presentation and twins) have been quite stable over time. Even though the prevalence of some of these indications may have increased, the increases are too small to explain the large increase in the Caesarean section rate. Thus, it is generally believed that some of the increase can be accounted for by non-medical factors. One potential factor to consider is the role of the funding system, and whether the funding system provides incentives in favour of Caesarean sections.

Most of the research in this area is from the USA. There physicians or hospitals are reimbursed from private and public insurance on a fee-for-service basis for each delivery. Studies have also been carried out in other countries. Many of these countries also have private funding. Within this field of research the focus has been on how relative differences in fees for Caesarean delivery and normal delivery can influence the choice of type of delivery (for example see: [23-29]). If the level of reimbursement for Caesarean delivery is high, this can influence physicians to choose Caesarean delivery rather than normal delivery. The empirical literature gives no clear answer to this research question. Some studies have found that higher fees for Caesarean delivery stimulate a higher rate of this type of delivery [25,28]. Other studies have found little or no effect [24,26,27]. Another type of study has focussed on the effect of remuneration systems and competition on the type of delivery [27-34]. One of the most cited studies is the study of Gruber & Owings (1996) [30]. During the period 1970 to 1982, the birth rate in the USA fell by 13.5 per cent. In order to prevent a fall in revenue, obstetricians compensated by carrying out more Caesarean sections, which generate more revenue than ordinary deliveries. Several other studies from the 1980s and the 1990s also found that the rate of Caesarean section was influenced by how physicians were remunerated [28,30,32].

To our knowledge, there are few studies that have investigated why the Caesarean section rate is also increasing in countries with publically-financed maternity care. This is a relevant research question, since the Caesarean section rates in these countries has also increased during the last decades. In this study, we investigated the increase in the Caesarean section rate in Norway. The organization of maternity care in Norway is quite different from countries with private funding. In Norway, women give birth in publically-owned and publically-financed hospitals [35]. Doctors receive a fixed salary and have no personal economic advantage by carrying out a Caesarean delivery rather than a normal delivery. There is little competition between hospitals for women giving birth. The country is divided into hospital areas in which the capacity of maternity units is planned according to the expected number of births within the catchment area. Mothers pay no fee, irrespective of the type of delivery. The hospitals receive revenue according to the number of deliveries. The revenue scheme is designed so that there is no economic advantage for the hospital to perform a Caesarean section rather than a normal delivery (for a detailed review of the organization of Norwegian hospitals, see [36]).

Over the last few decades, hospital budgets have increased markedly. For example, in Norway, the revenue per bed increased by almost 200 per cent from 1980 to 1990 (values adjusted for inflation) [35]. Similar increases have been reported from other OECD countries [37]. The aim of this study was to investigate whether the increase in hospital revenue has influenced the increase in the Caesarean section rate in Norway, and if it has, how large the effect is.

With increased budgets, the hospitals have greater opportunities to offer Caesarean deliveries, which are expensive, rather than normal deliveries, which are cheaper. There can be several non-medical explanations for doctors offering Caesarean deliveries rather than normal deliveries. The most common reason that is proposed is that the position of women in relation to obstetricians has been strengthened over time, and that it is therefore easier for women to have their wishes to have a Caesarean delivery met [38-40]. One consequence of this is that more Caesarean sections can be carried out as a result of pressure from mothers, even if a Caesarean delivery is not medically indicated. With increased hospital funding, it is easier for obstetricians to meet the wishes of these mothers. Another possible explanation is that the practice of obstetricians is influenced by fear of litigation if something goes wrong with a normal delivery [41-43]. In borderline cases, it is better to carry out a Caesarean section to be on the safe side. This issue has been studied particularly in the USA, where evidence to support this supposition has been found (for a review of the literature see: [41,43,44]). There are no Norwegian studies of defensive medicine in maternity care. However, it may be that some of the increase in the Caesarean section rate in Norway can be explained by the increase in hospital revenue giving more scope for obstetricians to practice defensive medicine. There have also been dramatic developments in medical technology, with a concurrent marked increase in survival for new-born babies, who would not have survived earlier [45,46]. The benefit of much of this new medical technology is greater with a less stressful delivery, which a Caesarean delivery can be.

We investigated the research question using a large set of data containing detailed medical information about all births in all hospitals in Norway during the period 1976-2005. We have information about annual revenue for each hospital. As far as we know, no-one has previously studied the effect of hospital revenue on choice of type of delivery over such a long period of time. The data and the analyses are described in detail in the Methods section. We then present the results. Finally we discuss our findings.

## Methods

### The source of the data

The analyses were carried out on data from the Medical Birth Registry (MBRN) of Norway for the period 1976-2005 http://www.fhi.no and data from Statistics Norway. In Norway, all maternity units have a duty to report all births to MBRN, i.e. the register encompasses the whole population of mothers who give birth [47]. The MBRN contains detailed information about the health status of the mother and the baby. The quality of these data are monitored regularly, and they are reliable [47,48]. The data from MBRN were aggregated to the level of the hospital, and merged with data about hospital revenue, which was obtained from Statistics Norway. There are 19 counties in Norway, which had responsibility for hospital funding during the period 1976-2001. From 2002 hospital ownership was transferred to five state-owned Regional Health Authorities [49]. Although hospital ownership changed, the system for allocating funding to hospitals did not change. This means that the revenue data are comparable for the whole period. The study has been approved by the Regional Committee for Medical and Health Research Ethics.

### The model specification

We estimated the following fixed effect regression model:

(1) $$\begin{array}{ll} \text{Caesa}{\text{r}}_{\text{it}} & =\alpha_{\text{i}}+\;\gamma \text{Year}{}_{\text{t}}+\;\beta_{\text{1}}\text{Rev}\text{\_}\text{hos}{\text{p}}_{\text{it}-\text{1}}\;\\ & +\;\beta_{\text{2}}\text{Control variable}{\text{s}}_{\text{it}}+\;\varepsilon_{\text{it}}\;\\ & \; \end{array}$$

where the subscript *i *denotes the hospital and *t *denotes year. *Caesar *is the Caesarean section rate for each hospital. Our key variable is *Rev_hosp*, which measures hospital revenue per bed in NOK 1000, deflated to 1970 prices by using the price index for local government consumption [50]. The variable is lagged one year; i.e. we assume that the present year's Caesarean section rate is determined by the previous year's hospital revenue per bed (for variable definitions and descriptive statistics see Table 1). Both *Caesar *and *Rev_hosp *were transformed into natural logarithms. In this log-log specification the estimated coefficient of the variable *Rev_hosp *gives the elasticity of the Caesarean section rate with respect to hospital revenue per bed.

**Table 1:** Variable definitions and descriptive statistics.

		Mean		
Variable	Definition	1976	Whole material	2005
caesar	= the proportion of Caesarean sections	0.047	0.108	0.146
		(0.028)	(0.044)	(0.049)
rev_hosp	= hospital revenue per bed in NOK 1000, deflated to 1970 prices	101.32	205.42	364.43
		(20.25)	(91.41)	(66.99)
age_le20^1^	= the proportion of mothers less than 20 years	0.116	0.057	0.026
		(0.033)	(0.033)	(0.013)
age_hi35^1^	= the proportion of mothers older than 35 years	0.044	0.072	0.117
		(0.015)	(0.031)	(0.030)
edu_univ^2^	= the proportion of mothers with university/college education	0.126	0.305	0.450
		(0.037)	(0.101)	(0.107)
edu_uss^2^	= the proportion of mothers with upper secondary school education	0.557	0.453	0.362
		(0.056)	(0.073)	(0.076)
weight_le2500^3^	= the proportion of babies with a birthweight less than 2500 g	0.045	0.034	0.031
		(0.016)	(0.020)	(0.021)
weight_hi4500^3^	= the proportion of babies with a birthweight greater than 4500 g	0.031	0.039	0.042
		(0.011)	(0.013)	(0.013)
ab_present	= the proportion of babies with abnormal presentation (including breech	0.043	0.059	0.088
	presentation, transverse presentation, abnormal cephalic presentation and other)	(0.016)	(0.024)	(0.030)
preeclam	= the proportion of mothers with preeclampsia (including unspecified, mild and severe)	0.021	0.029	0.031
		(0.111)	(0.015)	(0.016)
mult_birth	= the proportion of mothers with multiple births	0.025	0.024	0.025
		(0.010)	(0.024)	(0.019)
no_births	= the number of births in total	838	1028	1176
		(723)	(1034)	(1388)
weekend	= the proportion of births on a Saturday or a Sunday	0.248	0.248	0.274
		(0.025)	(0.031)	(0.111)
year_2002	= 1 if year equals 2002 to 2005, 0 otherwise			

Mean values and standard deviation in brackets.

^1 ^Reference category: the proportion of mothers aged 20-35 years

^2 ^Reference category: the proportion of mothers with compulsory school education

^3 ^Reference category: the proportion of babies with a birthweight 2500-4500 g

In Eq. (1), α_i _is the hospital-specific effect that varies *between *hospitals, but which does not vary for the *same *hospital over time. Therefore, α_i _measures all observable characteristics that are time-invariant for each hospital over time. In that way unobserved hospital-specific characteristics, which vary cross-sectionally between hospitals, are cancelled out. This is an advantage as this reduces potential bias in the regression coefficient for hospital revenue [51]. γ is a vector of year dummies, included to take account of events that can vary from year to year, but which affect all hospitals equally. ε_it _is an identically and independently distributed error term.

The distribution of the number of deliveries was very skewed (Table 2). A small number of hospitals had a very large number of deliveries. For example, the three largest hospitals have over 20 per cent of all births. This represented over 3 000 births on average per year. In comparison, the 20 smallest hospitals had only 13 per cent of all births. This represents less than 500 births on average per year. It is probable that measurement errors in the data are positively correlated with the number of births per hospital. Therefore, we have estimated Eq. (1) with the number of births per hospital as a weight.

**Table 2:** The number of hospitals according to the number of births per year. 1976-2005.

Number of births per year	Number of hospitals	Percentage of hospitals	Average number of births per year for all the hospitals within the category	Percentage of births
< 300	7	15.2	1 248	2.6
300-399	7	15.2	2 345	5.0
400-499	6	13.0	2 763	5.9
500-999	9	19.6	6 165	13.1
1000-1999	10	21.7	14 052	29.8
2000-2999	4	8.7	9 372	19.8
> = 3000	3	6.5	11 273	23.9

Total	46	99.9	47 218	100.1

### Control variables

Important control variables are characteristics of the health status of the mother and child, which vary between hospitals and over time [52,53]. Several of the medical conditions mentioned below are correlated with slow or no progress in labour or signs of foetal distress. A Caesarean section can then be indicated to prevent damage to the child. Large babies are more often delivered by Caesarean section than babies of average weight. The probability for a Caesarean section also increases if the foetus has an abnormal presentation, if preeclampsia is a complication, and if the mother has multiple births. Mothers with a high level of education have healthier babies than mothers with a low level of education [46,54]. This is taken account of by including the mothers' educational status in the analyses. We also included two variables that describe characteristics of the hospital: the number of births in total, and the proportion of births on a Saturday or a Sunday. A large maternity unit will have more experience and competence and better technology to deal with complicated deliveries than a small unit. Therefore, units with a high level of competence can carry out more complicated deliveries as normal deliveries instead of by Caesarean section. The result can be that there are relatively fewer Caesarean sections in large units than in small units [55,56]. It can also be expected that maternity units will try to carry out planned Caesarean deliveries on week days rather than weekends, as this is a way for them to save money. All control variables were log transformed. This was done in order to improve the interpretability of the regression coefficients. The regression coefficient then measure the percentage change in the Caesarean section rate by a one per cent change in an independent variable.

We did two additional analyses where we tested for trends and variations across variables. In the first analyses we included an interaction term between hospital revenue per bed (lagged) and number of births. There we tested whether the effect of hospital revenue varied according to the size of the hospital. In the second analysis we included interaction terms between hospital revenue per bed (lagged) and years - altogether 28 interaction terms. There we tested whether the effect of hospital revenue varied over time.

From 2002, hospital ownership changed. An assumption underlying our analysis is that the revenue data are comparable over time, i.e. that these data are not influenced by the change in ownership. This is tested by comparing the regression coefficient for the dummy variable for 2002 with the coefficient for 2001 (a pre- and post-2002 test). The effect of hospital ownership on hospital revenue data may appear with a lag. Therefore, we did further analyses by including separate dummy variables for the years successive to 2002.

### Number of observations

The analyses were carried out using data from 46 hospitals. We had data for hospital revenue for all the 46 hospitals for each year. Since hospital revenue was lagged for one year, the analyses were performed over a period of 29 years (1977-2005). This provided 1 334 observations over the 29-year period. We lacked information for some of the control variables for some of the hospitals for some years. A hospital is excluded from the analyses for the particular year where we lacked information for the control variables, i.e. the analyses were performed on an unbalanced set of data with 1 260 observations. In order to test the robustness of our results we did an additional analysis where we imputed values for missing data. For each control variable we calculated the mean values for each year. A missing value for a control variable for a hospital was replaced by the mean value for that variable for that particular year.

## Results

### Descriptive results

**Figure 1:**
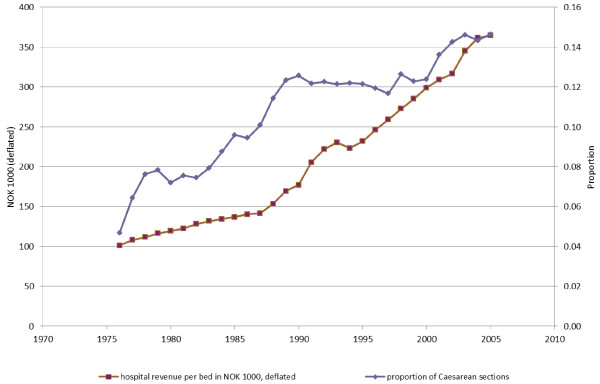
**Trends in hospital revenue per bed and Caesarean sections**.

Rates for all variables are reported in Table 1 and commented on below. The denominator in the rates is the number of deliveries per hospital. Both the Caesarean section rate for each hospital and hospital revenue per bed increased markedly during the 30-year period (Table 1 and Figure 1). The Caesarean section rate increased from 0.047 in 1976 to 0.146 in 2005. Correspondingly, hospital revenue per bed increased from just over NOK 100 000 to NOK 364 000 (adjusted for inflation). There was a slight increase in the mean number of births per hospital, from 838 in 1976 to 1176 in 2005. However, this increase is small in relation to the increase in the number of Caesarean sections.

There has also been an increase in some of the risk factors for Caesarean section. In particular, the proportion of babies with an abnormal presentation increased from 0.043 in 1976 to 0.088 in 2005. Also, the proportion of mothers with preeclampsia increased during the 30-year study period, from 0.021 in 1976 to 0.031 in 2005. The proportion of babies with a birth weight greater than 4500 g also increased slightly.

### Regression analysis - main results

The elasticity for the Caesarean section rate with respect to hospital revenue per bed was 0.137 (p < 0.05) (Table 3 column I). This implies that when hospital revenue per bed increases by 1 per cent then the Caesarean section rate increases by 0.137 per cent. From 1976 to 2005, hospital revenue increased by 260 per cent (Table 1). This represents an increase of about 35 per cent in the Caesarean section rate, if the elasticity from the estimations is used (the figure appears by multiplying 0.137 by 260). If we use the Caesarean section rate in 1976, which was 4.7 per cent, an increase of 35 per cent would predict that the Caesarean section rate in 2005 would be 6.4 per cent.

**Table 3:** Regressions with the proportion of Caesarean sections per hospital as the dependent variable.

Variable	Main analysis		Imputation for missing values		Test for whether the effect of hospital revenue per bed varies according to number of births	
	I		II		III	
Intercept	-1.262	**	-1.207	**	-3.210	**
	(2.38)		(2.45)		(3.12)	
rev_hosp (lagged)	0.137	**	0.153	**	0.462	**
	(2.38)		(2.63)		(2.88)	
age_le20^1^	-0.012		0.014		-0.016	
	(0.36)		(0.42)		(0.48)	
age_hi35^1^	0.111	**	0.123	**	0.110	**
	(2.78)		(3.12)		(2.77)	
edu_univ^2^	-0.064		0.015		-0.094	
	(0.78)		(0.20)		(1.13)	
edu_uss^2^	-0.372	**	-0.361	**	-0.424	***
	(2.97)		(2.88)		(3.34)	
weight_le2500^3^	-0.013		-0.009		-0.002	
	(0.53)		(0.39)		(0.11)	
weight_hi4500^3^	0.052		0.036		0.053	
	(1.59)		(1.12)		(1.61)	
ab_present	0.316	****	0.316	****	0.300	****
	(10.80)		(10.81)		(9.95)	
preeclam	0.126	****	0.122	****	0.130	****
	(6.57)		(6.34)		(6.77)	
mult_birth	1.709	*	1.715	*	1.283	
	(1.89)		(1.90)		(1.39)	
no_births	-0.122	**	-0.106	**	0.153	
	(3.06)		(2.65)		(1.15)	
weekend	-0.345	***	-0.261	**	-0.333	**
	(3.34)		(2.92)		(3.23)	
rev_hosp (lagged) × no_births					-0.046	**
					(2.17)	
						
Number of observations	1260		1334		1260	
						
Number of hospitals	46		46		46	
						
R^2^	0.74		0.74		0.74	

All variables ln transformed. Regression coefficients with t-values in brackets.

^1 ^Reference category: the proportion of mothers aged 20-35 years

^2 ^Reference category: the proportion of mothers with compulsory school education

^3 ^Reference category: the proportion of babies with a birthweight 2500-4500 g

**** p < 0.0001

*** p < 0.001

** p < 0.05

* p < 0.10

Most of the variables measuring characteristics of the health status of the mother and child have the expected effect on the Caesarean section rate. The Caesarean section rate increases by 0.052 per cent when the proportion of babies with a birthweight greater than 4500 g increases by 1 per cent (Table 3 column I). When the proportion of mothers 35 years and older increases by 1 per cent, the Caesarean section rate increases by 0.111 per cent. Further, when the proportion of babies with abnormal presentation increases by one per cent, then the Caesarean section rate increases by 0.316 per cent. From 1976 until 2005, the proportion of babies with abnormal presentation increased from 4.3 per cent to 8.8 per cent, that is an increase of 105 per cent (Table 1). This corresponds to an increase of 32 per cent in the Caesarean section rate. If we base our calculations on the Caesarean section rate in 1976, which was 4.7 per cent, an increase of 32 per cent would predict that the Caesarean section rate in 2005 would be 6.3 per cent. A similar calculation for the proportion of mothers who have preeclampsia predicts the Caesarean section rate to be 5.0 per cent by the end of our study period. The Caesarean section rate decreases by 0.122 per cent when the size of maternity units increases by 1 per cent (Table 3 column I). Also, the Caesarean section rate decreases by 0.345 per cent when the proportion of deliveries that are carried out at weekends increases by 1 per cent.

### Additional analyses

The results from the analysis where we imputed values for missing data for the control variables are presented in Table 3, column II. For nearly all variables the regression coefficients are almost identical to the coefficients for the main analysis (column I). The interaction term between hospital revenue per bed (lagged) and number of births is negative, and statistically significant at the conventional level (p < 0.05) (Table 3, column III). The sign of the coefficient implies that the effect of hospital revenue on the Caesarean section rate decreases as the hospital size increases. The interaction terms between hospital revenue per bed and years are not statistically significant at conventional levels (F-value = 1.47; p > 0.05). The difference between the regression coefficients for 2002 and 2001 is 0.0052, with a t-value of 0.12 (p = 0.90). The fact that the difference between these coefficients is not statistically different from zero lends support to the assumption that the revenue data are comparable over time. None of the differences between the coefficient for 2001 and the coefficients for the years successive to 2002 were statistically significantly different from zero at conventional levels (p < 0.05). This indicates that the effect of changes in hospital ownership on hospital revenue data does not appear with a lag.

## Discussion

This is the first study in which the effect of hospital funding on the number of Caesarean sections has been analysed over such a long period of time. In Norway, as in most western countries, funding of hospitals has increased markedly from the 1970s up to the present day. This increase has led to only a minor increase in Caesarean sections. Therefore, an increase in hospitals' revenue per bed does not seem to have led to mothers more often having their desire to have a Caesarean section as the preferred mode of delivery met, and/or that obstetricians practice more defensive medicine. This indicates that choice of mode of delivery is mainly determined by medical criteria.

Our finding is in accordance with another recently published study from Norway which shows that the position of mothers in maternity care has been weakened over time [57]. In fact, the positions of the obstetricians have been strengthened due to the introduction of advanced diagnostic technology, such as ultrasound, cardiotocography, ST wave-form analysis and foetal blood analyses. These new technologies have reduced clinical uncertainty, so that obstetricians are less dependent on judgement and interpretation of information from mothers for assessing whether the delivery is progressing without complications. This has reduced the possibility for mothers to influence their choice of delivery.

It is likely that the increased funding to hospitals has improved the quality of maternity care, for example by employing more qualified personnel, and by investing in medical equipment that reduces the risk of complications during delivery. In particular, the advances in diagnostic technology have improved foetal monitoring, both before and during delivery (for an overview of different technologies see: [58]). With better diagnosis and monitoring, it is also easier to detect foetal asphyxia and slow progress in labour, which are often indications for a Caesarean section. Concurrent with the increase in hospital funding, with the subsequent increase in the Caesarean section rate, a marked decrease in infant mortality has been observed in Norway [59,60]. Annual mean infant mortality, measured as deaths under 1 year of age per 1000 live births, was 9.0 for the period 1976-1980 and 3.5 for the period 2001-2005 [60]. A corresponding decrease has been observed in most of the other OECD countries [61]. A contributory factor to this decrease can be the raised standard of both personnel and equipment that has resulted from increased hospital funding [45].

There was a slight increase in several of the risk factors during the study period (Table 1). For example, the babies became heavier and the mothers became older. There was also an increase in the proportion of babies with abnormal presentation and mothers with preeclampsia. All these risk factors had a positive effect on the Caesarean section rate. Our calculations above showed that the predicted Caesarean section rate on the basis of the risk factors is far less than the observed rate of 0.146 in 2005. This supports the view of epidemiologists that the increase in the Caesarean section rate during the last three decades is considerably greater than could be expected, based on risk factors alone [19-22]_. _Our results also support findings from other studies, which show that units with a high level of competence can carry out more complicated deliveries as normal deliveries instead of Caesarean section [55,56]. This is particularly the case for hospitals that have high revenue per bed (Table 3, column III). These hospitals have a sufficient number of personnel and advanced technology to deal with normal deliveries that are complicated, and which otherwise would have been delivered by Caesarean section. That fewer Caesarean sections are carried out at weekends than on weekdays is as expected. Hospitals save money in this way.

From a policy point of view the findings of our study are encouraging. Caesarean sections are mainly determined by the medical risk factors of the mother and the baby. An increase in hospital revenue can be used by policy makers as an instrument to increase the quality of maternity services both in terms of more qualified personnel and equipment. Increased hospital budgets do not lead to any major side-effects such as unnecessary Caesarean sections, either because the obstetricians meet the wishes of the mothers for type of delivery, or because the obstetricians practice defensive medicine.

Caution must be used in generalizing the findings of this study to other countries where maternity care is organized differently. This applies particularly to countries where many births take place in private clinics, where obstetricians are remunerated on a fee-for-service basis and where mothers have private insurance for deliveries. For example, if obstetricians are remunerated on a fee-for-service basis, there can be an incentive to carry out a Caesarean section rather than a normal delivery (for a review see: [62]). In that case the obstetricians might go along with the mother's wish for a Caesarean section because this is consistent with the obstetrician's own private economic interests. Similarly, in health care systems with private health insurance the obstetricians can perform Caesarean sections that are not medically necessary in order to avoid litigation if something goes wrong with a normal delivery.

## Conclusion

In conclusion, our findings show that the effect of an increase in hospital revenue per bed on the Caesarean section rate in Norway during the last three decades is small as indicated by an elasticity of 0.13. The strength of our study is that we have estimated a cause and effect relationship. This has been done in three different ways. First, we used a fixed effect model in which unobserved hospital-specific characteristics, which vary cross-sectionally between hospitals, have been cancelled out. Second, we used lagged revenue; i.e. the revenue variable is measured one year ahead of the Caesarean section rate. Third, in the analyses we have included an extensive set of control variables for the risk factors of the mother and the baby. These risk factors are correlated with revenue, and they vary between hospitals over time. These variables have significant effects on the Caesarean section rate. The inclusion of variables for the risk factors of the mother and the baby increases the likelihood that we have obtained an unbiased and causal estimate of the effect that hospital revenue per bed (lagged) has on the Caesarean section rate.

From a policy perspective hospital budgets can be increased without that leading to any side-effects such as unnecessary Caesarean sections either because the obstetricians meet the wishes of the mothers about type of delivery, or because the obstetricians practice defensive medicine. Rather, increased hospital revenue is likely to improve the quality of maternity care.

## Competing interests

The authors declare that they have no competing interests.

## Authors' contributions

JG and RS conceived the study, planned its design and its coordination, and drafted the manuscript. IS organized and built the database, and LM performed the statistical analyses. TH organized all the hospital data, and contributed to data interpretation. AE arranged the funding, and critically revised the content of the manuscript. All authors approved the final version of the manuscript.

## Pre-publication history

The pre-publication history for this paper can be accessed here:

http://www.biomedcentral.com/1472-6963/11/267/prepub
